# Dr. Blaikie Smith

**Published:** 1909-04-03

**Authors:** 


A Reuter telegram from San Remo, dated March 26, announces the death from heart-failure of Dr. Blaikie Smith, a Scottish medical practitioner residing there. Dr. Blaikie Smith graduated at Aberdeen in 1878 with honours, and held appointments in the Medical School and University of that city.

